# Anaphylaxis to Celebrex during an oral challenge

**DOI:** 10.1186/1710-1492-10-S1-A38

**Published:** 2014-03-03

**Authors:** Vaishaali Manga, Gordon Sussman

**Affiliations:** 1 Department of Internal Medicine, Memorial University, St. John’s, Newfoundland and Labrador, A1B3V6, Canada; 2 Department of Clinical Immunology and Allergy, University of Toronto, Toronto, Ontario M4V 1R2, Canada

## Case

We report a case of a 65-year-old female with an anaphylactic type reaction during a graded oral celecoxib challenge. She was previously using NSAIDS and Celebrex (celecoxib) for analgesia in 2005. However, due to the side effect profile of COX2 inhibitors, Celebrex was discontinued. The patient’s primary analgesic was ibuprofen. Upon reintroduction of Celebrex, the patient developed urticaria after the second dose and urticaria with angioedema after the third dose. When the patient presented to the allergy clinic, it was felt that the urticaria and angioedema were secondary to ibuprofen. Because she had a negative crude prick skin test to Celebrex, an oral challenge to Celebrex was done. One hour after the 200 mg cumulative challenge, the patient was asymptomatic and an additional 200 mg was given. Twenty minutes later the patient developed facial flushing, angioedema, respiratory distress and hypotension, with her blood pressure dropping to 78/53 mmHg from a baseline of 120/80 mmHg. The patient was treated with epinephrine 0.1 mg subcutaneously, without recovery. After 5 and 15 minutes additional 0.2 mg subcutaneous epinephrine injections were administered. She was also treated with Reactine 20mg and prednisone 50mg. The patient was stabilized within thirty minutes.

The patient was able to tolerate ibuprofen and sulfonamide antibiotics without any side effects. She was also taking hydrochlorothiazide on a daily basis for hypertension. She does not have any previously documented allergies or adverse reactions to medications.

## Discussion

Our patient had an anaphylactic type reaction to celecoxib. Most patients that have a reaction to celecoxib are thought to have a hypersensitivity to the sulfonamide moiety. There are two types of sulfonamide moieties in medications. Antibiotics that contain sulfonamide have the arylamine moiety, whereas other medications such as hydrochlorothiazide contain the nonarylamine moiety. Usually patients with a hypersensitivity to the arylamine sulfonamide can tolerate medications with the nonarylamine sulfonamide, as they do not typically cross react. In our case, the patient tolerated sulfonamide antibiotics (arylamine) and hydrochlorothiazide (nonarylamine) meaning that she is not reacting to either of the sulfonamide groups, but rather another component of Celebrex.

## Conclusion

Anaphylaxis to Celebrex, although rare, has been reported in the literature. We report a case of anaphylactic reaction to Celebrex in a patient that tolerated both arylamines and nonarylamines sulfonamides, suggesting that our patient has a hypersensitivity to a different component of Celebrex. This requires further study and demonstrates necessary caution when challenging with COX2 inhibitors.

